# Signaling Governed by G Proteins and cAMP Is Crucial for Growth, Secondary Metabolism and Sexual Development in *Fusarium fujikuroi*

**DOI:** 10.1371/journal.pone.0058185

**Published:** 2013-02-28

**Authors:** Lena Studt, Hans-Ulrich Humpf, Bettina Tudzynski

**Affiliations:** 1 Institut für Lebensmittelchemie, Westfälische Wilhelms-Universität, Münster, Germany; 2 Institut für Biologie und Biotechnologie der Pflanzen, Westfälische Wilhelms-Universität, Münster, Germany; University of Wisconsin – Madison, United States of America

## Abstract

The plant-pathogenic fungus *Fusarium fujikuroi* is a notorious rice pathogen causing hyper-elongation of infected plants due to the production of gibberellic acids (GAs). In addition to GAs, *F. fujikuroi* produces a wide range of other secondary metabolites, such as fusarins, fusaric acid or the red polyketides bikaverins and fusarubins. The recent availability of the fungal genome sequence for this species has revealed the potential of many more putative secondary metabolite gene clusters whose products remain to be identified. However, the complex regulation of secondary metabolism is far from being understood. Here we studied the impact of the heterotrimeric G protein and the cAMP-mediated signaling network, including the regulatory subunits of the cAMP-dependent protein kinase (PKA), to study their effect on colony morphology, sexual development and regulation of bikaverins, fusarubins and GAs. We demonstrated that fusarubin biosynthesis is negatively regulated by at least two Gα subunits, FfG1 and FfG3, which both function as stimulators of the adenylyl cyclase FfAC. Surprisingly, the primary downstream target of the adenylyl cyclase, the PKA, is not involved in the regulation of fusarubins, suggesting that additional, yet unidentified, cAMP-binding protein(s) exist. In contrast, bikaverin biosynthesis is significantly reduced in *ffg1* and *ffg3* deletion mutants and positively regulated by FfAC and FfPKA1, while GA biosynthesis depends on the active FfAC and FfPKA2 in an FfG1- and FfG3-independent manner. In addition, we provide evidence that G Protein-mediated/cAMP signaling is important for growth in *F. fujikuroi* because deletion of *ffg3*, *ffac* and *ffpka1* resulted in impaired growth on minimal and rich media. Finally, sexual crosses of *ffg1* mutants showed the importance of a functional FfG1 protein for development of perithecia in the mating strain that carries the MAT1-1 idiomorph.

## Introduction

Members of the genus *Fusarium* are known to produce a wide range of secondary metabolites, such as mycotoxins and pigments, under defined environmental conditions. This ability makes them a promising tool to study regulatory aspects of secondary metabolite production at the molecular level. The heterothallic ascomycete *Fusarium fujikuroi* (teleomorph: *Gibberella fujikuroi*) was first described as the causative agent of the ‘bakanae’ or foolish seedling disease of rice plants due to the production of gibberellic acids (GAs), isoprenoid phytohormones [1]–[3]. Infected rice plants show hyper-elongated internodes, chlorotic leaves, sterile or empty grains and they finally die during late stages of infection [4]. Besides GAs, *F. fujikuroi* is able to produce various other secondary metabolites such as the mycotoxins fusarin C [5], fusaric acid [6] and fumonisins [7], the red polyketide bikaverin [8] or the terpenoid α-acorenol [9]. Moreover, the recently sequenced genome of the *F. fujikuroi* wild-type strain IMI58289 revealed the presence of further putative secondary metabolite gene clusters the products of which remain to be identified (B. Tudzynski and Co-workers, unpublished data). However, their regulation appears to be quite diverse and little understood.

A prerequisite for the initiation of secondary metabolite biosynthesis under specific conditions is the ability to sense and transduce external signals to downstream targets, *e.g*. transcription factors, which in turn can activate the expression of genes involved in biosynthesis of a certain secondary metabolite. Prominent components of signal transduction pathways are heterotrimeric G proteins that integrate a variety of signals and transduce them to downstream signaling cascades.

In general, heterotrimeric G proteins are composed of a Gα subunit and a tightly associated dimer of β and γ subunits (Gβγ). The Gα subunit is associated with a seven transmembrane helices-containing G protein-coupled receptor (GPCR) at the plasmamembrane [10]. In the inactive state Gα is bound to GDP. Once the GPCR is activated by its cognate ligand, conformational changes in the receptor induce the exchange of GDP for GTP leading to its activation and subsequent dissociation from the Gβγ dimer. Both the activated Gα subunit and the dissociated Gβγ subunits are now able to bind and regulate downstream effectors [11]. One well known downstream effector of the activated Gα is the adenylyl cyclase that produces the second messenger cyclic AMP (cAMP) which in turn binds to the regulatory subunits of the cAMP-dependent protein kinase A (PKA). In general, filamentous fungi possess one regulatory subunit and two catalytic subunits of PKA. In the inactive state two regulatory subunits are tightly associated with two catalytic subunits. Upon cAMP-binding to the regulatory subunits the affinity for the catalytic subunits decreases. Subsequently the catalytic subunits of the PKA are released and are now able to phosphorylate downstream targets [12], [13]. As G proteins constitute the first components of signaling chains they represent a promising target to study the involvement of such signaling cascades in cellular processes including secondary metabolism.

Most filamentous fungi have three Gα proteins, each belonging to classes I, II or III [12]. Gα subunits of class I show high sequence similarity and are known to be involved in the regulation of fungal development, pathogenicity and secondary metabolism. The best studied example with regard to secondary metabolism is FadA from *Aspergillus nidulans*. Deletion of *fadA* resulted in upregulation of sterigmatocystin and aflatoxin biosynthesis, while constitutive activation of *fadA* resulted in precocious sterigmatocystin production as well as increased penicillin biosynthesis [14]–[16]. Furthermore, alterations in secondary metabolism have been observed in Gα mutants from *Botrytis cinerea* [17], *Cryphonectria parasitica* [18], *F. graminearum* [19], *F. sporotrichioides* [16], *Neurospora crassa* [20], *Penicillium chrysogenum* [21], *P. marneffei* [22] and *Trichoderma atroviride* [23].

Contrary to Gα proteins from class I, Gα subunits belonging to classes II and III have so far not been linked to secondary metabolism in filamentous fungi. Although Gα subunits of class III are clearly involved in fungal development and pathogenicity (*e.g*. *A. nidulans*, *B. cinerea* and *F. graminearum*) [24]–[26], deletion of class II Gα proteins had only minor effects on fungal metabolism [27], [17].

First indication for the involvement of G protein signaling in secondary metabolism in *F. fujikuroi* was the observation that deletion of *ffg1* resulted in the secretion of a red pigment into the surrounding medium when grown on agar plates. To our surprise the red pigmentation could not be linked to bikaverin biosynthesis because expression of the bikaverin genes was not elevated in this mutant. Recently a second group of red pigments, the fusarubins, has been identified in *F. fujikuroi* [28]. These results encouraged us to study the involvement of G protein-mediated signaling and further downstream targets with the main focus on secondary metabolism. In this paper we show that FfG1 and FfAC, but neither of the PKAs regulates the formation of the perithecial pigments fusarubins, and that FfG1-mediated signaling is crucial for sexual development in *F. fujikuroi*. We provide evidence that both bikaverins and GAs are regulated via the cAMP/PKA signaling cascade and for the first time show the involvement of a class III Gα subunit in secondary metabolism.

## Materials and Methods

### Fungal strains and culture conditions

For all knock-out experiments either the wild-type strain *Fusarium fujikuroi* IMI58289 (Commonwealth Mycological Institute, Kew, United Kingdom) or *F. fujikuroi* MRC-1995 (kindly provided by J.F. Leslie, Kansas State University) was used. For cultivation in submerse culture *F. fujikuroi* strains were pre-incubated in 300 ml Erlenmeyer flasks with 100 ml Darken medium (DVK) [29] on a rotary shaker for 72 h at 28°C and 180 rpm. For RNA isolation and secondary metabolite analysis a 500 µl aliquot of this culture was used for inoculation of synthetic ICI (Imperial Chemical Industries Ltd., United Kingdom) medium [30] with the desired nitrogen source and cultivated for an additional 3–7 days. Protoplasts were produced in 100 ml ICI medium with 10 g/l fructose instead of glucose and 1 g/l (NH_4_)_2_SO_4_ using 500 µl of the pre-incubated culture and additional cultivation for more than 16 h on a rotary shaker at 28°C and 180 rpm. For the nitrogen shift-experiments the mycelia were grown in ICI media on a rotary shaker at 28°C and 180 rpm. After 4 days either 33 mM glutamine, 33 mM sodium nitrate or no nitrogen was added and cultures were incubated for additional 30 min. Plate assays were performed on complete medium (CM) as well as on Czapek Dox (CD) medium for up to 7 days [31], [32]. For sexual crosses the fungal strains were grown on carrot agar according to Klittich and Leslie [33]. For DNA isolation, the fungus was grown on solid CM with Cellophane overlays for 3 days at 28°C. For RNA isolation, the fungus was grown in ICI medium with the specified nitrogen source for 3–4 days at 28°C and 180 rpm upon pre-incubation in DVK.

### Plasmid constructions

Plasmid construction for deletion of *ffpka1* was accomplished by amplification of about 1 kb upstream and downstream sequences of *ffpka1* using the primer pairs ffpka1_5_SacII//ffpka1_5_XbaI (upstream) and ffpka1_3_SalI//ffpka1_3_XhoI (downstream) containing the following restriction sites: 5′ SacII/XbaI, 3′ SalI/XhoI. Subsequently the fragments were cloned into pNR1 which contains the nourseothricin resistance cassette [34]. Finally the knock-out fragment was restricted using SacII/XhoI and used for *F. fujikuroi* transformation. All other knock-out fragments subsequently used for *F. fujikuroi* transformation were created using yeast recombinational cloning [35]. The 5′ and 3′ regions of the corresponding genes were amplified with appropriate primer pairs, for the 5′ region, 5F and 5R primers were used, and for the 3′ region, primers 3F and 3R were used, work based on the genomic sequence available for *F. fujikuroi* IMI58289 (B. Tudzynski and co-workers, unpublished data). Hygromycin and nourseothricin were used as resistance markers. The hygromycin resistance cassette, consisting of the hygromycin B phosphotransferase gene *hph* [36], driven by the *trpC* promoter, was amplified using the primer pair hph-F/R from the template pCSN44. All primers used for PCR were obtained from Eurofins GmbH (Ebersberg, Germany) (table S1). The obtained fragments were cloned into the *Saccharomyces cerevisiae* strain FY834 together with the EcoRI/XhoI-restricted plasmid pRS426. For complementation of Δ*ffg1* and constitutive activation of FfG1 the gene *ffg1*, including ∼2-kb promoter and ∼200-bp terminator sequence, was amplified in two fragments using proof-reading polymerase with the primer pairs Com_Ga1_1F//Com_Ga1_1R and Com_Ga1_2F//Com_Ga1_2R. The FfG1^G42R^ primers used for amplification (DA_Ga1_1F//DA_Ga1_1R and DA_Ga1_2F//DA_Ga1_2R) contained base pair exchanges for the amino acid conversion (fig. S2). The obtained fragments were cloned into *S. cerevisiae* together with the SacI/SpeI restricted pNDN-OGG containing a nourseothricin resistance cassette [37]. Correct assembly of the plasmid was verified by sequencing. The same strategy was used for complementation of Δ*ffg3* and Δ*ffac* yielding vectors NDN-OGG-*ffg1^C^*, NDN-OGG-*ffg1* ^G42R^, NDN-OGG-*ffac^C^* and NDN-OGG-*ffg3^C^*, respectively (fig. S3).

### Standard molecular methods

For DNA isolation, lyophilized mycelium was ground to a fine powder in liquid N_2_ and prepared according to Cenis [38] and afterwards used for PCR amplification. For PCR, 25 ng genomic DNA, 5 pmol of each primer, 200 nM deoxynucleoside triphosphates, and 1 unit BioTherm™ DNA polymerase (GeneCraft GmbH, Lüdinghausen, Germany) was used for diagnostic PCR and amplification of upstream and downstream regions in yeast-recombinational cloning. PCRs were performed as follows: initial denaturating step at 94°C for 3 min followed by 35 cycles of 1 min at 94°C, 1 min at 56–60°C, 1–2 min at 70°C and a final elongation step at 70°C for 10 min. In order to amplify the knock-out constructs from yeast-derived plasmids, the TAKARA polymerase kit was used as indicated. For complementation strategies and constitutive activation of FfG1 the respective genes were amplified from genomic DNA of *F. fujikuroi* IMI28589 (B. Tudzynski and Co-workers, unpublished data) using a proof-reading polymerase. To this end, 25 ng genomic DNA, 5 pmol of each primer, and 1 unit of Phusion® polymerase (Finnzymes, Thermo Fisher Scientific, Finland) was used. Plasmid DNA from *S. cerevisiae* was extracted using the yeast plasmid isolation kit (SpeedPrep, DualsystemsBioTech) and directly used for PCR reaction. In case of complementation, the obtained vectors were cloned into *E*. *coli* and subsequently extracted using the GeneJETTM Plasmid Miniprep Kit (Fermentas GmbH, St. Leon-Rot, Germany) and sequenced using the BigDye® Terminator v3.1 Cycle Sequencing Kit and the ABI PRISM® 3730 Genetic Analyzer (Applied Biosystems, Foster City, CA, USA) according the manufacturer's instructions. DNA and protein sequence alignments were done with DNA STAR (Madison, WI, USA).

For northern blot analysis RNA was isolated from lyophilized mycelium. The mycelium was ground in liquid N_2_ and RNA was extracted using the RNAagents total RNA isolation kit (Promega, Mannheim, Germany) according to the manufacturer's instructions. An amount of 20 µg per sample was loaded on a 1% agarose gel and run under denaturing conditions (1% (v/v) formaldehyde) [39]. Afterwards, samples were transferred to Hybond-N^+^ membranes and hybridization was performed according to the method of Church and Gilbert [40]. Phylogenetic analyses were performed with the web-based tool at www.phylogeny.fr [41]. Microscopy of perithecia was performed using a SteREO Discovery. V20™ microscope equipped with an AxioCam MRc (Carl Zeiss MicroImaging GmbH, Jena, Germany).

### Fungal transformations

Protoplasts were prepared either from *F. fujikuroi* IMI58289, for deletion of *ffg1*, *ffg3*, *ffac*, *ffpka1* and *ffpka2*, or from *F. fujikuroi* MRC-1995, for deletion of *ffg1.* Transformation was carried out as previously described [42]. About 10^7^ protoplasts were transformed with ∼10 µg of the amplified replacement cassettes for generating the knock-outs. For complementation of Δ*ffg1* the deletion mutant Δ*ffg1_T9* was transformed with 10 µg of the plasmid pNDN-OGG-*ffg1^C^,* containing *ffg1* driven by its native promoter. According to this Δ*ffg3_T6* and Δ*ffac_T7* were complemented with 10 µg of the plasmids NDN-OGG-*ffg3^C^* and NDN-OGG-*ffac^C^*, respectively. For constitutive activation of FfG1 the *F. fujikuroi* wild-type strain IMI58289 was transformed with 10 µg of the plasmid NDN-OGG-*ffG1* ^G42R^. Transformed protoplasts were regenerated as described by Tudzynski *et al*. [43]. The medium contained the appropriate resistance marker. Single conidial cultures were established from either hygromycin B- or nourseothricin-resistant transformants and used for subsequent DNA isolation.

### Analysis of secondary metabolites

For analysis if bikaverin and fusarubins, culture fluids from 3-to 7-day old cultures were filtered through a 0.2 µm membrane filter (Millex®, Millipore) and directly used for analysis without further preparation, as described in Studt *et al*. [28]. For gibberellic acid analysis, aliquots (20 ml) of culture fluid from 7– day-old cultures were extracted over a C18-cartridge and measured using HPLC-UV as described by Wiemann *et al.* [44]. GA accumulation was quantified relative to the dry weight of the respective trains to exclude the possibility that merely impaired growth of an isolate was responsible for the observed effects on GA biosynthesis.

## Results

### Identification of Gα subunits in *Fusarium fujikuroi*

**Figure 1:**
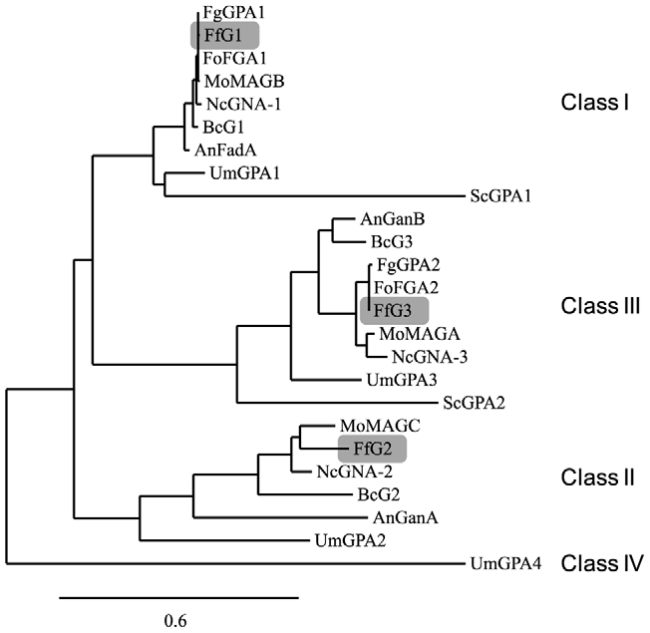
Phylogenetic relationship between *Fusarium fujikuroi* FfG1, FfG2, FfG3 and Gα proteins from other fungi. The phylogram was generated based on multiple sequence alignments [41] using the following G proteins: FadA, GanA and GanB from *Aspergillus nidulans* [15], [24]; BcG1, BcG2 and BcG3 from *Botrytis cinerea* [17], [26]; FfG1, FfG2, FfG3 from *Fusarium fujikuroi* (this paper); GPA1, GPA2 from *F. graminearum* [19]; FGA1, FGA2 from *F. oxysporum* [47], [49]; MAGB, MAGC and MAGA from *Magnaporthe oryzae* [27]; GNA-1, GNA-2 and GNA-3 from *Neurospora crassa* [46], [48]; GPA1 and GPA2 from *Saccharomyces cerevisiae* [86] and GPA1, GPA2, GPA3 and GPA4 from *Ustilago maydis* [87]. The Gα protein class IV GPA2 that is unique for *U. maydis* was used as out-group. The bar presents 0.6 character change. Organisms: An *Aspergillus nidulans*, Bc *Botrytis cinerea*, Ff *Fusarium fujikuroi*, Fg *F. graminearum*, Fo *F. oxysporum*, Mo *Magnaporthe grisea*, Nc *Neurospora crassa*, Sc *Saccharomyces cerevisiae* and Um *U. maydis*.

Like most other filamentous fungi *F. fujikuroi* possesses three Gα subunits, designated as FfG1 (AJ315470), FfG2 (HF563556) and FfG3 (HF563557). These three proteins were identified in the genome of the *F. fujikuroi* wild-type strain IMI58289 by a BlastP search [45] using the protein sequences of BCG1 (GenBank: CCD53386.1), BCG2 (GenBank: CCD44342.1) and BCG3 (GenBank: CCD47156.1) from *Botrytis cinerea* as a reference [17], [26]. Gα proteins from different fungi, including *F. fujikuroi*, can be divided into three main classes based on amino acid sequence similarities: FfG1 groups together with homologues in other fungi into class I, e.g. FadA from *A. nidulans* [46], MAGB from *M. oryzae* [27], GNA-1 from *N. crassa* [47], FGA1 from *F. oxysporum* [48] and GPA1 from F. *graminearum* [19]. This class of Gα proteins shares sequence similarities with the mammalian Gα_i_ proteins that are thought to inhibit adenylyl cyclase activity [12]. FfG3 grouped together with homologues in class III, *e.g*. GanB from *A. nidulans* [24], MAGA from *M. oryzae* [27], GNA-3 from *N. crassa* [49] and GPA2 from *F. graminearum* [50] all showing high similarity with mammalian Gα_s_ which stimulate adenylyl cyclase activity [12]. Finally, FfG2 belongs to the fungal Gα class II which has no mammalian counterpart [12] (fig. 1).

### FfG1 functions as a negative regulator for fusarubin biosynthesis

In order to study the functions of FfG1 in *F. fujikuroi* the gene was deleted by homologous integration of a hygromycin resistance cassette into the wild-type strain IMI 58289, hereafter called wild type (WT). Homologous integration of the resistance cassette was verified by diagnostic PCR (fig. S1A). Two independent deletion mutants, Δ*ffg1-T9* and Δ*ffg1-T13*, were obtained that show identical phenotypes. Hence, Δ*ffg1-T9* was arbitrarily chosen for further investigations. In addition to *ffg1* deletion mutants, we generated transformants expressing a constitutively active version of the Gα subunit FfG1 with an exchange of glycine for arginine at position 42 (FfG1^G42R^) in the WT background. This mutation was previously shown to prevent the intrinsic GTPase activity of Gα and thereby the inactivation of the heterotrimeric G protein [46]. The transformation and subsequent characterization of nourseothricin-resistant transformants yielded three independent transformants (*ffg1* ^G42R^) (fig. S2). Integration of the vector and presence of point mutation was verified by diagnostic PCR and subsequent sequencing of the obtained PCR fragments (data not shown).

**Figure 2:**
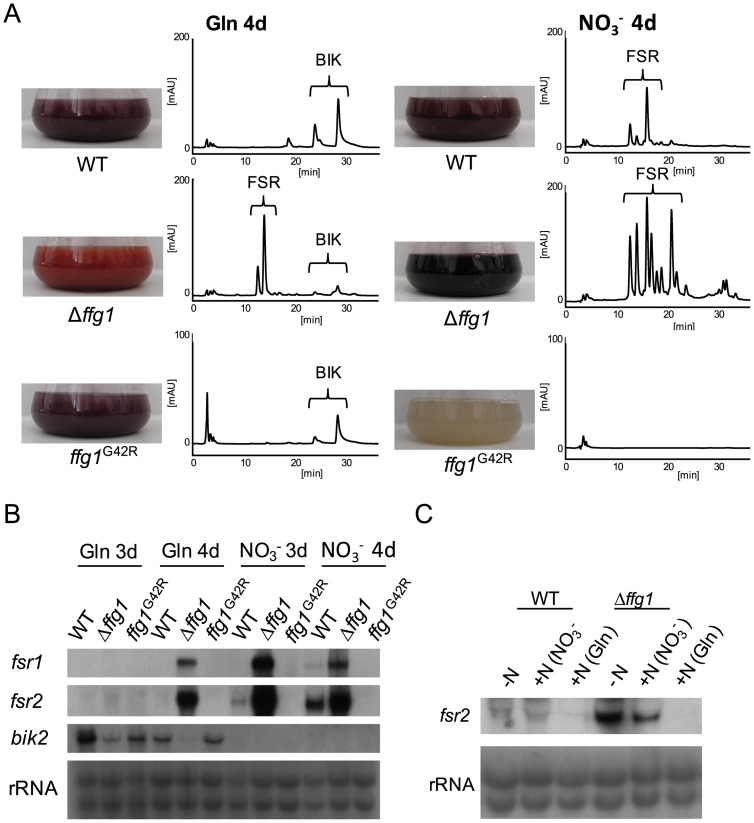
FfG1 has distinct functions as a repressor for fusarubin biosynthesis. The WT, Δ*ffG1* and *ffG1* ^G42R^ mutants were grown in ICI synthetic media either under fusarubin biosynthesis-repressing (6 mM glutamine (Gln)) or fusarubin biosynthesis-favoring (sodium nitrate (NO_3_^−^)) conditions. A) After 7 days of incubation, culture filtrates were used for HPLC-DAD analysis (for details see material and methods). Photographs show the flask with the culture broth after 7 days of growth and the corresponding HPLC chromatogram at 450 nm. Brackets indicate metabolites found in the liquid culture: bikaverins (BIK) and fusarubins (FSR). The scale for *ffG1* ^G42R^ was set to 20 mAU due to a lower accumulation of compounds in the liquid culture compared to the other strains. B) After 3 and 4 days, mycelia were harvested and used for northern blot analysis. The genes *fsr1* and *fsr2* (as examples for fusarubin biosynthetic genes) and *bik2* (as an example for bikaverin biosynthetic genes) were used as probes. C) WT and Δ*ffg1* were grown under fusarubin biosynthesis-favoring conditions. After 4 days of growth either glutamine, sodium nitrate or no nitrogen was added and after another 30 min mycelia were harvested and used for Northern blot analysis, *fsr2* was used for probing.

To study the impact of *ffg1* mutations on secondary metabolism we grew the wild type and the Δ*ffg1* and the *ffg1* ^G42R^ mutants in synthetic ICI medium with either 6 mM glutamine or 6 mM sodium nitrate, representing optimal culture conditions for the production of the two red polyketides, bikaverins and fusarubins, respectively [51], [52], [28]. Using sodium nitrate as sole nitrogen source both the wild type and the deletion mutant showed the typical dark red pigmentation characteristic for the accumulation of fusarubins (fig. 2A). Using glutamine, the coloration of the culture broth of Δ*ffg1* differed markedly from that of the wild type. Subsequent analysis using high performance liquid chromatography (HPLC) coupled to a diode array detector (HPLC-DAD) revealed that the Δ*ffg1* mutant produced only trace amounts of bikaverin in contrast to the wild type. However, high concentrations of fusarubins, not present in the wild type under these conditions, were produced in Δ*ffg1* (fig. 2A). Northern blot analysis verified the HPLC data as fusarubin biosynthetic genes (*fsr1* encoding the polyketide synthase; *fsr2* encoding an *O*-methyltransferase) were up-regulated in Δ*ffg1* under both fusarubin-favoring (6 mM sodium nitrate) and bikaverin-favoring (6 mM glutamine) conditions (fig. 2B).

In the *ffg1* ^G42R^ mutant, no expression of fusarubin genes and no fusarubin production has been detected under both conditions (fig. 2A–B). Surprisingly, the *ffg1* ^G42R^ mutant produced also less bikaverin under bikaverin-favoring conditions, and, accordingly, bikaverin biosynthetic genes were significantly down-regulated. Under repressing conditions (6 mM sodium nitrate) the bikaverin genes were not expressed (fig. 2B). Neither deletion nor constitutive activation of *ffg1* ^G42R^ showed any alterations with regard to GA biosynthesis or colony morphology (data not shown).

To investigate whether deletion of *ffg1* also results in de-regulation of fusarubin biosynthesis under nitrogen excess, glutamine (33 mM), sodium nitrate (33 mM) or water (no nitrogen) was added to cultures which were grown for four days under nitrogen limiting conditions (6 mM sodium nitrate). Cultures were incubated for an additional 30 min and analyzed for changes in gene expression. Fusarubin biosynthetic genes (*fsr2*) are up-regulated under nitrogen starvation as shown previously and upon additions of sodium nitrate (fig. 2B). However, repression of fusarubin biosynthetic genes by glutamine excess could not be overruled in the *ffg1* deletion mutant compared to the wild type (fig. 2C). Complementation of *ffg1* deletion mutants by homologous reintroduction of the respective gene, driven by its native promoter, rescued the wild-type phenotype with regard to fusarubin and bikaverin biosynthesis (fig. S3/fig. S4).

### Functional characterization of further components of the cAMP pathway

A prominent downstream target of heterotrimeric G proteins is the adenylyl cyclase. The activated enzyme produces cAMP which in turn activates the PKA via binding to its regulatory subunit [11]. In order to find out if FfG1 regulates the fusarubin biosynthesis via the cAMP/PKA signaling pathway, we studied the single components of this pathway in more detail. The adenylyl cyclase sequence as well as the two catalytic subunits of the PKA were identified by BlastP analyses [45] in the genome database of *F. fujikuroi* IMI58289 (B. Tudzynski and co-workers, unpublished data) using the corresponding protein sequences of *B. cinerea* BAC [53], BcPKA1 and BcPKA2 [32] as query. Accordingly, the *F. fujikuroi* proteins were designated as FfAC (HF563555), FfPKA1 (HF563558) and FfPKA2 (HF563559). All genes were deleted in the *F. fujikuroi* wild type. Three independent deletion mutants of *ffac* were obtained Δ*ffac_T7,* Δ*ffac_T12* and Δ*ffac_T14* (fig. S1C). As all three mutants showed the same phenotypes Δ*ffac_T7* was arbitrarily chosen for further studies. Deletion of *ffpka1* yielded three independent deletion mutants: Δ*ffpka1*_*T4*, Δ*ffpka1_T10*, Δ*ffpka1_T15*, and deletion of *ffpka2* resulted in four independent mutants: Δ*ffpka2*_*T10*, Δ*ffpka2_T12*, Δ*ffpka2_T15* and Δ*ffpka1*_*T16*. As all Δ*ffpka1* mutants on the one hand, and all Δ*ffpka2* on the other hand, showed a similar phenotype, Δ*ffpka1*_*T4* and Δ*ffpka2*_*T6* were arbitrarily chosen for further investigation (fig. S1D–C).

### The cAMP/PKA pathway has impact on secondary metabolism

**Figure 3:**
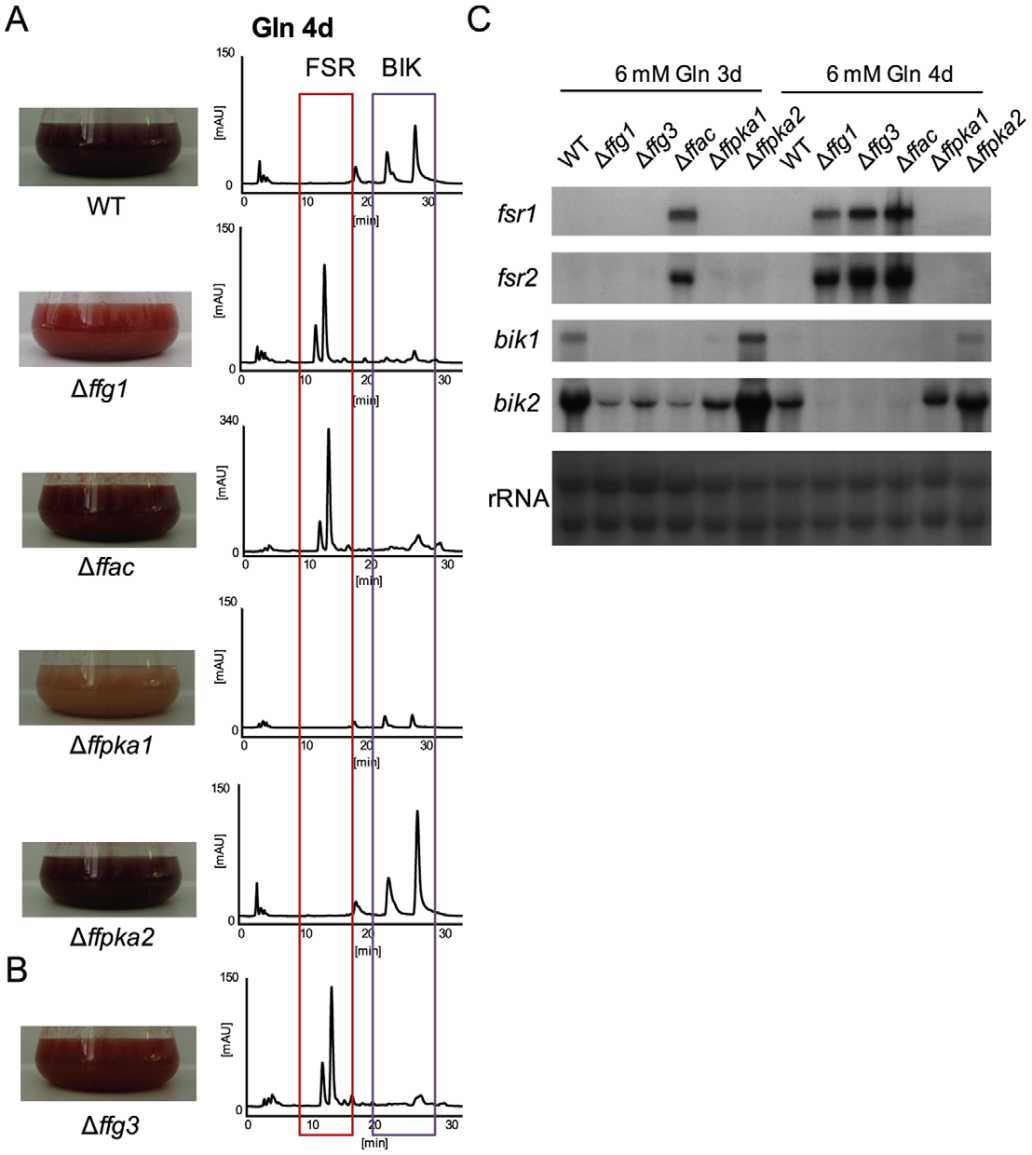
Deletion of *ffg1, ffac* and *ffg3* results in fusarubin biosynthesis under unfavorable conditions. The WT and cAMP pathway mutants (Δ*ffg1,* Δ*ffac,* Δ*ffpka1,* Δ*ffpka2,* Δ*ffg3*) were grown for 3, 4 (for Northern blot analysis) or 7 days (for chemical analysis) in synthetic ICI medium (6 mM glutamine (Gln)). A) Liquid cultures were directly used for HPLC-DAD analysis (for details see material and methods). Fusarubins (FSR) are highlighted by the red box, bikaverins (BIK) by the purple box. Flasks containing culture broth of the WT and cAMP pathway mutants (Δ*ffg1,* Δ*ffac,* Δ*ffpka1,* Δ*ffpka2*) after 7 days of cultivation are shown on the left, corresponding HPLC-DAD chromatograms at 450 nm on the right. The scale for Δ*ffac* was set to 340 mAU due to a higher accumulation of compounds compared to the other strains. B) Photograph of the culture broth and corresponding HPLC-DAD chromatogram at 450 nm of Δ*ffg3*. C) Northern blot of WT and cAMP pathway mutants after 3 and 4 days of growth under fusarubin unfavorable conditions. The fusarubin biosynthetic genes, *fsr1* and *fsr2*, and the bikaverin biosynthetic genes, *bik1* and *bik2*, were used for probing.

Similarly to Δ*ffg1*, deletion of *ffac* also caused significant up-regulation of fusarubin biosynthesis under favoring (6 mM sodium nitrate) (data not shown) as well as under non-favoring conditions (6 mM glutamine), while bikaverin biosynthesis is decreased in both conditions (fig. 3A). Surprisingly, neither deletion of *ffpka1* nor *ffpka2* induced the formation of fusarubin. However, bikaverin production was reduced in the Δ*ffpka1* but not the Δ*ffpka2* mutant (fig. 3A). It is notable that deletion of *ffac* resulted in an about three times higher accumulation of fusarubins compared to the Δ*ffg1* mutants under both fusarubin-favoring (data not shown) and non-favoring conditions (fig. 3A). Expression of fusarubin-biosynthetic genes in Δ*ffac* mutants started earlier, already after 3 days of incubation, while expression in Δ*ffg1* mutants was detectable only after 4 days (fig. 3C). The much stronger effect of FfAC on fusarubin biosynthesis, compared to that of the upstream-acting FfG1, indicates the existence of an additional upstream activator of FfAC besides FfG1 exist. In several filamentous fungi two of the three Gα subunits (class I and III) were shown to stimulate the AC activity [11], [12]. For example, in *B. cinerea* addition of exogenous cAMP can partially restore the defects of the respective knock-out mutants [17], [53], [26]. To test whether FfG3, a Gα proteins belonging to class III, is also involved in fusarubin regulation we analyzed the *ffg3* deletion mutants in more detail. Indeed, we found elevated fusarubin gene expression and a strong accumulation of these pigments under non-favoring conditions indicating the involvement of FfG3 in fusarubin biosynthesis, as it was shown for FfG1 and FfAC (fig. 3). Complementation of Δ*ffac* and Δ*ffg3* deletion mutants by homologous reintroduction of the respective gene, driven by its native promoter, restored the wild-type phenotype with regard to fusarubin biosynthesis (fig. S3).

### FfVEL1 and FfG1 regulate pigment biosynthesis in an opposite manner

**Figure 4:**
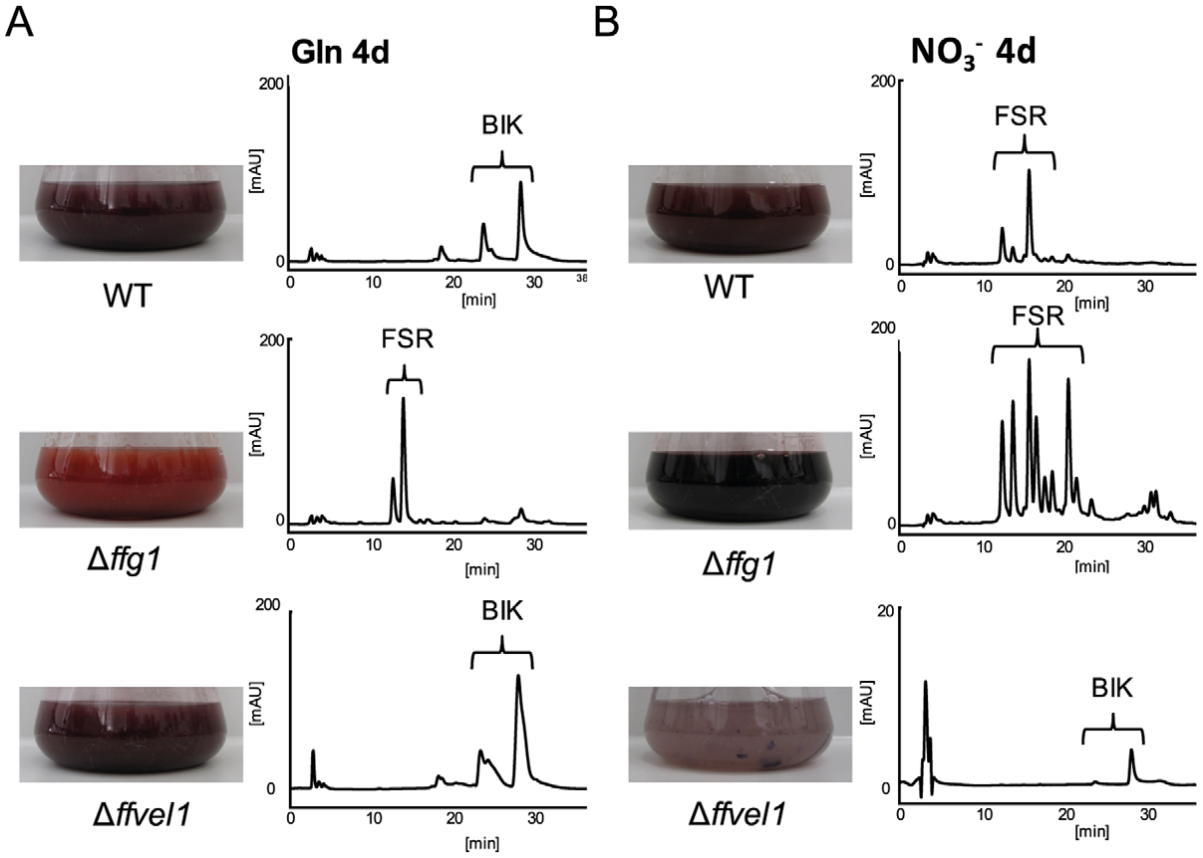
Opposite regulation of pigments by FfVEL1 and FfG1. The WT, Δ*ffg1* and *Δffvel1* were grown in ICI synthetic media in either A) bikaverin biosynthesis-favoring (6 mM glutamine (Gln)) or B) fusarubin biosynthesis-favoring (sodium nitrate (NO_3_^−^)) conditions for 3, 4 (for Northern blot analysis) or 7 days (for HPLC analysis). Liquid culture fluid was filtered and used for HPLC-DAD analysis (for details see material & methods). Photograph of flasks containing culture broth of the indicated strains after 7 days of growth and the corresponding HPLC chromatogram at 450 nm. Brackets indicate group of metabolites found in the liquid culture: bikaverins (BIK) and fusarubins (FSR). The scale for Δ*ffvel1* was set to 20 mAU due to a lower accumulation of compounds compared to the other strains.

Previously we have shown that the global regulator Velvet negatively regulates bikaverin production in *F. fujikuroi*: bikaverin biosynthetic genes are dramatically upregulated in the Δ*ffvel1* mutant under inducing conditions [54]. Even under conditions unfavorable for bikaverin production small amounts of bikaverin can be detected in the liquid medium. Studying the impact of the *ffvel1* deletion on fusarubin biosynthesis, we observed a strong inhibition under both conditions (fig. 4). Therefore, deletion of *ffg1* caused opposing effects compared to the deletion of *ffvel1*: less bikaverin, but increased formation of fusarubins under inducing, as well as repressing, conditions in contrast to the elevated bikaverin and reduced fusarubin production in the Δ*ffvel1* mutant (fig. 4).

### GA biosynthesis is regulated by FfAC and FfPKA2 and does not involve FfG1 and FFG3

**Figure 5:**
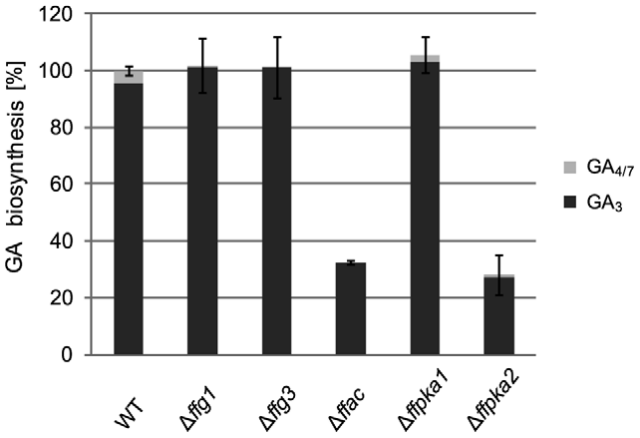
Gibberellic acid (GA) content of cAMP pathway mutants compared to WT. The WT and cAMP pathway mutants (Δ*ffg1,* Δ*ffg3,* Δ*ffac,* Δ*ffpka1,* Δ*ffpka2*) were grown for 7 days in synthetic ICI medium under GA biosynthesis-favoring conditions (6 mM glutamine). GA_3_ and GA_4/7_ were extracted from 20 ml liquid culture (for details see material and methods). For comparison, GA content produced by the WT was set to 100%. Experiments were performed in triplicate.

GA biosynthesis is the ‘trade mark’ of *F. fujikuroi*, as only a few fungal species are able to produce these isoprenoid phytohormones. Therefore, we wanted to know if biosynthesis of GAs is also regulated via the G protein/cAMP pathway. We cultivated the wild type and mutant strains under GA biosynthesis-inducing conditions (6 mM glutamine) for 7 days. The quantity of produced GAs was analyzed by HPLC-UV. Interestingly, neither of the two Gα subunits, FfG1 and FfG3, was involved in regulation of GA biosynthesis. However, deletions of *ffac* and *ffpka2* resulted in a significant reduction of GA biosynthesis of about 70% (fig. 5), while deletion of *ffpka1* had no effect. These data indicate that FfAC can be activated by additional components other than the two Gα subunits, and that the two catalytic subunits of the PKA have distinct functions.

### Involvement of G protein-mediated cAMP/PKA signaling in fungal growth

**Figure 6:**
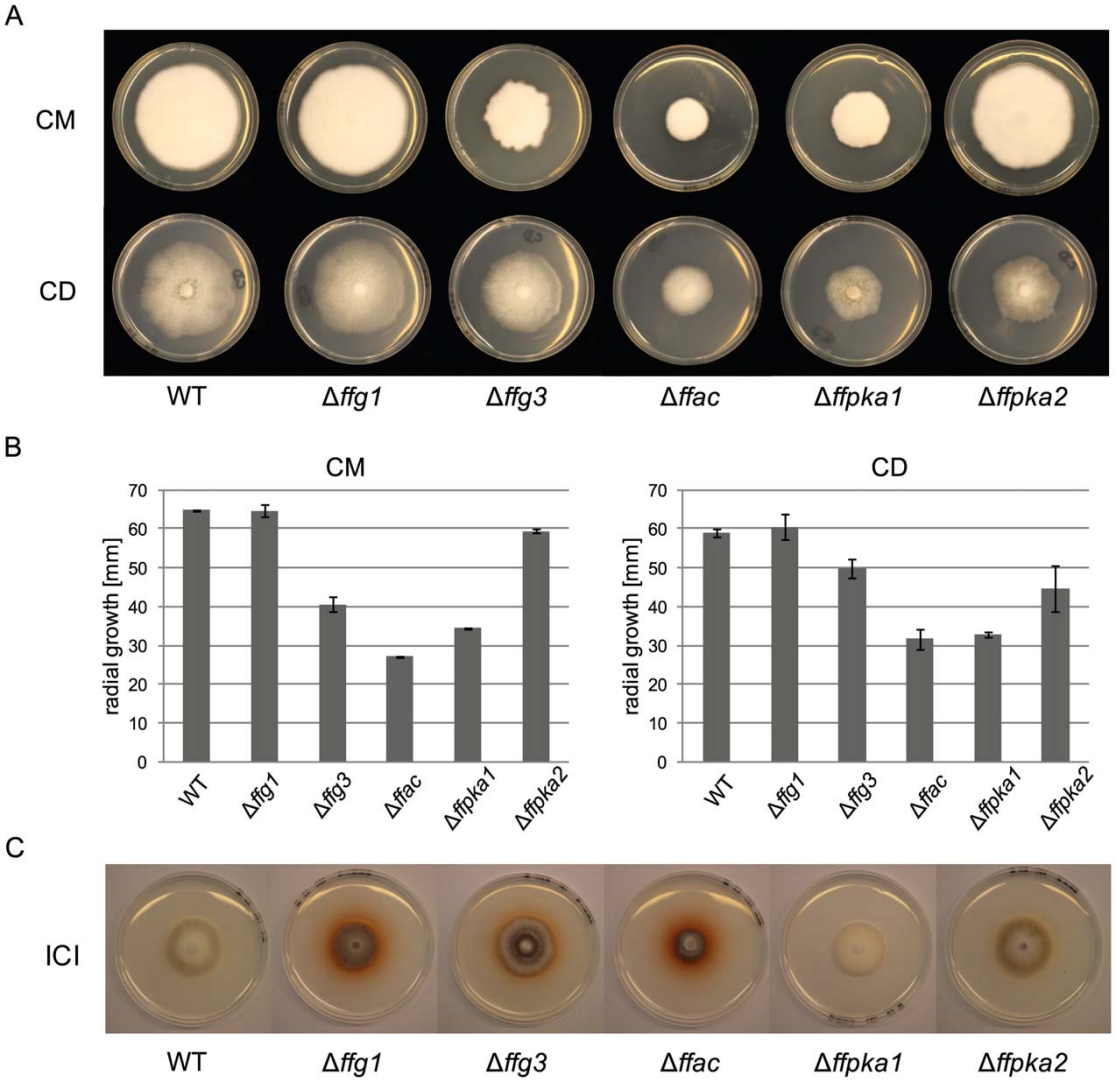
Analysis of growth behavior of the cAMP pathway mutants on different media compared to the WT. A) WT and cAMP pathway mutants (Δ*ffg1,* Δ*ffg3,* Δ*ffac,* Δ*ffpka1,* Δ*ffpka2*) were grown on solidified ICI medium with 6 mM sodium nitrate as sole nitrogen source. B) WT and cAMP pathway mutants on solidified complete medium (CM) and Czapek dox (CD, minimal medium). After 7 days radial growth rates of the respective mutants was determined. Experiments were done in triplicate. C) Radial growth of the respective mutants grown on CM or CD medium was measured [mm]. Mean values and standard deviations are shown in the diagram.

To study the involvement of the analyzed signaling components on fungal growth in *F. fujikuroi*, we grew the wild type and the different mutants for 7 days on both complete medium (CM) and minimal Czapek-Dox (CD) medium and determined the radial growth rates. When grown on CM, Δ*ffg3*, Δ*ffac* and Δ*ffpka1* showed reduced growth rates, while no growth defects were observed for Δ*ffg1* and Δ*ffpka2* (fig 6A–B). The same tendency was observed when the indicated strains were grown on CD medium. Although Δ*ffg1* grows in a wild type manner, Δ*ffg3*, Δ*ffac* and Δ*ffpka1* mutants showed impaired growth. Surprisingly, while Δ*ffpka2* showed normal growth on complete medium, radial growth was reduced about 25% on minimal medium (fig. 6A–B). When grown on synthetic ICI medium, Δ*ffg1*, Δ*ffg3*, and Δ*ffac* mutants secreted fusarubin into the medium, emphasizing our findings that the two Gα subunits and the adenylyl cyclase negatively affect fusarubin biosynthesis (fig. 6C). No significant differences were observed in liquid ICI medium regarding biomass accumulation of the single deletion mutants.

### FfG1 affects sexual development

Deletion of several class I Gα subunits have been shown to affect the sexual development, resulting e.g. in female sterility in *Cryphonectria parasitica, N. crassa*, and *F. graminearum*, and loss of perithecial formation in *M. grisea* [55], [56], [18], [57], [19], [27]. Recently we have shown that fusarubins are the pigments in perithecia in *F. fujikuroi* [28]. As biosynthesis of these perithecial pigments is upregulated in Δ*ffg1* we wished to determine whether FfG1 is also involved in the regulation of sexual development in *F. fujikuroi*.

**Figure 7:**
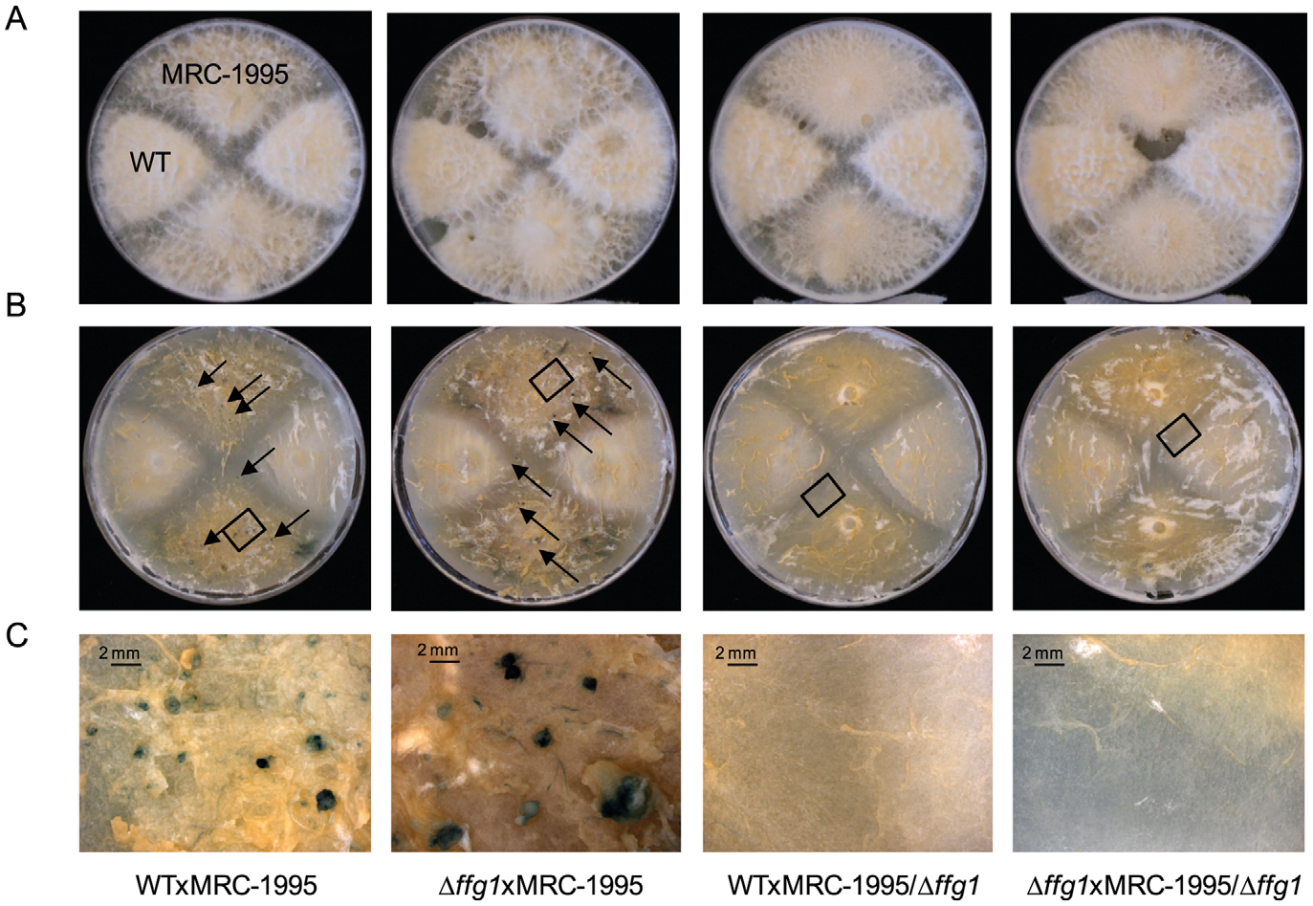
Loss of FfG1 affects perithecial formation. Strains were crossed as described in Materials and Methods. Crosses from left to right were WTxMRC-1995, Δ*ffg1*xMRC-1995, WTxMRC-1995/Δ*ffg1*, Δ*ffg1*xMRC-1995/Δ*ffg1*. A) Photographs of crosses after 10 weeks. B) Aerial hyphae were removed prior to microscopy to uncover the fruiting bodies (indicated by black arrows). C) Enlargement of segments high-lighted by black boxes in B). Perithecia are formed during crosses of WTxMRC-1995 and Δ*ffg1*xMRC-1995, but not in WTxMRC-1995/Δ*ffg1*, Δ*ffg1*xMRC-1995/Δ*ffg1*. Size standards are shown in the lower left corners.

Therefore, *ffg1* was also deleted in the mating partner of strain IMI58289, *F. fujikuroi* MRC-1995, carrying the MAT1-1 idiomorph, using the same targeted deletion strategy. Two independent deletion mutants were obtained, designated MRC-1995/Δ*ffg1*_*T44* and MRC-1995/Δ*ffg1*_*T49* (fig. S1F). Crosses were made between both wild types, one of either wild-type strains and the Δ*ffg1* mutant of the respective mating partner as well as between the two Δ*ffg1* mutant strains. When *ffg1* is only deleted in *F. fujikuroi* IMI58289 that carries the MAT1-2 idiomorph, perithecia were normally produced in a cross with MRC-1995, similarly to the cross between the two wild type strains. However, *ffg1* deletion in *F. fujikuroi* MRC-1995 carrying the MAT1-1 idiomorph resulted in total loss of perithecial formation in a cross with IMI58289, as it was the case when *ffg1* was deleted in both mating partners. (fig. 7).

## Discussion

### The Gα subunits FfG1 and FfG3 negatively regulate fusarubin biosynthesis

Deletion of *ffg1* resulted in an increased accumulation of fusarubin under both fusarubin-inducing and -repressing conditions, while constitutive activation of FfG1 (FfG1^G42R^) completely abolished fusarubin production under either of the investigated conditions. Bikaverin biosynthesis was decreased under both activating and repressing conditions (fig. 2 A–B), indicating that both pigments are regulated in opposing ways, and that fusarubin biosynthesis requires inactivation of the FfG1-dependent signaling pathway.

The involvement of class I Gα proteins in secondary metabolism has been studied in a variety of filamentous fungi. Interestingly, Gα proteins of class I share sequence similarities with mammalian Gα_i_ that are thought to inhibit adenylyl cyclase activity [12]. However, an increasing number of reports indicate that this class of Gα subunits has an activating effect on the adenylyl cyclase activity in filamentous fungi (*e.g.* in *M. oryzea*, *N. crassa* or *B. cinerea*) [27], [57], [17], [53], [26]. In general, there are several examples demonstrating that members of class I Gα subunits are involved in the regulation of secondary metabolism. For example, constitutive activation of PGA1 in *P. chrysogenum* resulted in increased production of three secondary metabolites: penicillin, the yellow pigment chrysogenin and the mycotoxin roquefortine [21]. Constitutive activation of FadA in *F. sporotrichioides* led to elevated T2-toxin levels [16], and constitutive active GasA^G42R^ in *P. marneffei* transformants showed an increased pigment production [22]. In contrast, constitutive activation of GNA-1 in *N. crassa* resulted in decreased carotenoid biosynthesis [20]. Deletion of Gα resulted in reduced pigmentation in *C. parasitica* [18] and increased deoxynivalenol and zearalenone production in *F. graminearum* [19]. Clearly, there are examples for activation as well as repression of secondary metabolite biosynthesis through G protein-mediated signaling. Even within the same species distinct secondary metabolites might be differentially regulated by Gα subunits. For example, in *A. nidulans* inhibition of *fadA* is necessary for the activation of sterigmatocystin and aflatoxin biosynthesis [14], [15] while penicillin biosynthesis requires the activation of FadA [16]. The same contradictory relationship has been found in *T. atroviride* in which the Gα subunit Tga1 has opposite roles regarding the regulation of the biosynthesis of different antifungal substances [23] indicating the complexity of G protein-mediated regulation of fungal secondary metabolism.

Interestingly, deletion of *ffac* resulted in a more severe de-regulation of fusarubin biosynthesis than deletion of *ffg1* indicating the involvement of at least one further upstream activator of *ffac*. In *B. cinerea*, two Gα subunits, BcG1 (class I) and BcG3 (class III), are involved in the activation of the adenylyl cyclase BAC. Deletion of *bcg1* and *bcg3* resulted in reduced cAMP levels; and respective phenotypes i.e. colony morphology and conidial germination could be restored by exogenous cAMP [17], [53], [26]. Gα proteins of class III have been shown to play crucial roles in fungal development and pathogenesis in various fungi [27], [48], [58], [59], but so far no connection between these Gα proteins and secondary metabolism has been observed. In *F. fujikuroi* deletion of *ffg3* indeed resulted in a similar phenotype to that of Δ*ffg1* providing strong evidence that both *ffg3* and *ffg1* independently activate FfAC. This model would explain the much stronger phenotype of the Δ*ffac* mutant regarding fusarubin biosynthesis.

### The second messenger cAMP, but not PKA, inhibits fusarubin biosynthesis in *F. fujikuroi*

Beside Gα subunits, the adenylyl cyclase and cAMP/PKA-signaling pathway in general have been shown to be involved in secondary metabolism in some fungi including *A. nidulans* [60], [61], [15], *T. virens* [62], *F. verticillioides* [63], [64] and *F. proliferatum* [65]. Recently the adenylyl cyclase of *F. fujikuroi* has been characterized in strain MRC-1995 [66]. The results presented here for the deletion of the corresponding gene in *F. fujikuroi* IMI58289 resembled those obtained for strain MRC-1995. Both deletion mutants showed impaired growth and decreased production of GA_3_. Furthermore, the authors described the over-production of a reddish pigment secreted into the agar which was not observed in the wild type under the same condition. However, the chemical structure of this red pigment remained unclear in this work [66]. Here, we show for the first time that the recently identified fusarubins in *F. fujikuroi* are identical with the reddish pigments secreted into the medium by Δ*ffac* [28] (fig. 3A). This assumption is further strengthened by the recent detection of fusarubin biosynthesis in the *F. fujikuroi* wild-type strain MRC-1995 [67].

Earlier publications on adenylyl cyclase mutants in *F. proliferatum* and *F. verticilliodes* showed similar results: an overproduction of a reddish pigment which was interpreted as an up-regulation of bikaverin biosynthesis [65], [63]. The results obtained during this study suggest that the observed reddish pigmentation in *F. verticillioides*, as well as *F. proliferatum*, is due to the production of fusarubins instead of bikaverin as it has been shown for *F. fujikuroi*. The presence of a fusarubin gene cluster has now been verified also in *F. verticillioides* [68] and *F. proliferatum* (B. Tudzynski and Co-workers, unpublished data) supporting our hypothesis that the released pigments are fusarubins, and not bikaverin.

It is well known that cAMP activates the PKA thus generating a functional link between the adenylyl cyclase and the PKA in the so called cAMP-dependent signaling pathway. Therefore, similar effects would be expected from a deletion of the adenylyl cyclase-encoding gene and deletions of genes encoding the catalytic subunits of the PKA. Surprisingly, neither deletion of *ffpka1* nor deletion of *ffpka2* resulted in a phenotype similar to that of the Δ*ffac* mutant because they did not produce fusarubins under repressing conditions (fig. 3A). These data suggest that other so far unknown cAMP-binding proteins, beside the regulatory subunit of the PKA, exist that might be involved in the regulation of fusarubin biosynthesis. Similar uncoupled functions of the AC and the PKA have been described in other fungi, although so far not linked to secondary metabolism (e.g. *B. cinerea*, *M. grisea* and *S. sclerotiorum*) [26], [53], [32], [69], [70], [71].

### Bikaverin biosynthesis is dependent on cAMP/PKA signaling

While formation of fusarubin was significantly increased, bikaverin biosynthesis was strongly decreased in Δ*ffg1* mutants (fig. 2) suggesting that FfG1 is also involved in regulation of bikaverin. However, bikaverin biosynthesis is neither increased under inducing- nor under repressing-conditions in the mutant with the constitutive active FfG1^G42R^, indicating that bikaverin is not a direct target of FfG1 in *F. fujikuroi* (fig. 2 A–B). Furthermore, deletion of *ffg3*, *ffac* and *pka1* also resulted in transcriptional downregulation of bikaverin genes and reduced bikaverin production. A recently generated *ffpka1* overexpression mutant revealed an elevated expression of *bik1* (Y. Eilert and B. Tudzynski, unpublished data). These results indicate that bikaverin biosynthesis in *F. fujikuroi* is directly regulated by FfAC and FfPKA1, and indirectly by the Gα subunits FfG1 and FfG3 due to their stimulating effects on the adenylyl cyclase.

### GA biosynthesis depends on cAMP and PKA, but does not involve FfG1 or FfG3

GA biosynthesis was found to be positively affected by FfAC [66; this paper], but neither deletion of *ffg1* nor *ffg3* resulted in a similar phenotype (fig. 5). These data strongly suggest that an additional upstream activator of FfAC is involved in the regulation of GA biosynthesis in *F. fujikuroi*. Small GTPases have been shown to stimulate the cAMP pathway in *S. cerevisiae* and *B. cinerea* [72], [32] and might therefore also be involved in activation of FfAC and subsequently in regulation of GA biosynthesis in *F. fujikuroi*. Furthermore, in *Candida albicans* and *Cryptococcus neoformans* the adenylyl cyclase is also activated by bicarbonate, thus combining the functionality of both mammalian adenylyl cyclase families [73]–[75]. Therefore, stimulation of FfAC due to changing CO_2_ levels might also be possible in *F. fujikuroi*. However, involvement of FfG2 cannot be excluded, although deletion of class II Gα proteins showed only minor effects on growth and development in other fungi (*e.g*. *B. cinerea*, *M. oryzea*) [17], [27].

Deletion of *ffpka2*, but not of *ffpka1*, resulted in reduced GA yields in a similar manner to the Δ*ffac* mutant indicating that FfPKA2 specifically functions as a positive regulator of GA biosynthesis since FfPKA1 can not compensate for the function of the missing FfPKA2 (fig. 5). So far investigations on homologues of FfPKA2 in other fungi revealed only a minor role in fungal development and virulence. For example, in *A. fumigatus* PKAC2 is involved in the regulation of germination, cell wall homeostasis and carbohydrate metabolism [76], while deletion of *PkaB* in *A. nidulans* and *bcpka2* in *B. cinerea* did not result in any phenotypic alterations [77], [32]. However, deletion of both catalytic subunits is lethal in *A. nidulans* [77], indicating that the catalytic subunits can at least partially complement each other. The significant reduction of GA formation in both the Δ*ffac* and Δ*ffpka2* mutants indicates specific functions of both PKA complexes. To our knowledge this is the first example of an independent role for PKA2 in secondary metabolism.

### Growth is regulated by FfG3, FfAC and FfPKA1 in *F. fujikuroi*

G proteins have been shown to be involved in several developmental processes, and deletion of several fungal class I Gα subunits have been shown to result in impaired growth (*e.g*. *A. nidulans*, *M. oryzea* and *N. crassa*) [45], [27], [57]. Furthermore, deletion of downstream targets of Gα showed similar phenotypes indicating the involvement of the adenylyl cyclase and PKA in the regulation of fungal growth in several fungal species, e.g. *A. nidulans* [60], [78], *A. fumigatus* [58], [79], *N. crassa* [80], *M. grisea* [69], *B. cinerea* [53], [32] and *F. verticillioides* [63]. In *A. nidulans*, constitutive activation of Gα (class I) also propagates the vegetative growth signal through the cAMP/PKA signaling pathway [81], [82], [60], [45].

In *F. fujikuroi*, deletion of *ffg1* did not result in an altered growth behavior, but deletion of *ffg3* caused reduced growth rates on minimal as well as on complete medium (fig. 6). Defects in colony morphology upon deletion Gα subunit class III have also been shown for *A. fumigatus*, *T. atroviride* and *C. parasitica* [58], [83], [18]. Radial growth was even more impaired in Δ*ffac* and Δ*ffpka1* compared to Δ*ffg3* indicating further upstream stimulators of FfAC. Notably, deletion of *ffpka2* showed normal growth rates on complete medium, while radial growth was impaired on minimal medium indicating a growth defect of Δ*ffpka2* likely due to a nutritional component that is included in the complete but missing in the minimal medium.

### Deletion of the class I Gα subunit FfG1 results in loss of fertility in the MRC-1995 genomic background

In heterothallic ascomycetes such as *F. fujikuroi* a single mating type locus, *MAT*, with two alternative forms (*MAT1-1*and *MAT1-2*) called mating types or idiomorphs, controls mating [84]. Sexual reproduction is initiated by the binding of pheromone peptides to the respective pheromone receptors, which are both regulated by MAT loci. By physical interaction of pheromone receptors, each coupled to a heterotrimeric G protein complex, G protein-mediated signaling is involved in fungal sexual reproduction in various organisms (*e.g*. *C. parasitica*, *N. crassa* or *F. graminearum*) [55], [56], [18], [57], [19], [11].

It was therefore anticipated that deletion of *ffg1* in *F. fujikuroi* would result in impaired sexual reproduction, but to our surprise only if deletion of *ffg1* was performed in the *F. fujikuroi* wild-type strain MRC-1995, carrying the MAT1-1 idiomorph. Deletion of *ffg1* in strain IMI58289, carrying the MAT1-2 idiomorph, showed no alterations in mating as purple perithecia were still developed (fig. 7). Recently, we have shown that the 4′phosphopantetheinyl transferase (PPTase), FfPpt1, is essentially involved in production of PKS- and NRPS-derived secondary metabolites, conidiation and sexual development. When the *Ffppt1* gene was missing in strain MRC-1995 carrying the MAT1-1 idiomorph, no recognition took place when contiguously grown with strain IMI58289 of the opposite mating type while deletion of *Ffppt1* in IMI58289 resulted in wild-type like formation of perithecia [44]. The reasons for mating type-dependent differences are not yet understood, but these examples provide strong evidence that the *MAT1-1* locus specifically regulates components which are essential for sexual reproduction. In the case of *ffppt1* deletion in the MAT1-1 background, sexual recognition of both partners was abolished, disclosing the possibility that the *MAT1-1* idiomorph specifically controls a PKS- and/or NRPS-derived metabolite or its receptor. However, recognition of the opposite mating partners still took place when *ffg1* was deleted in strain MRC-1995 as hyphae of opposite mating partners grew together (fig. 7A). This strengthens the assumption that G protein-mediated signaling is important for the middle and late stages of sexual reproduction but not for the early events, such as recognition of the respective mating partner as described earlier for *F. graminearum* [85].

**Figure 8:**
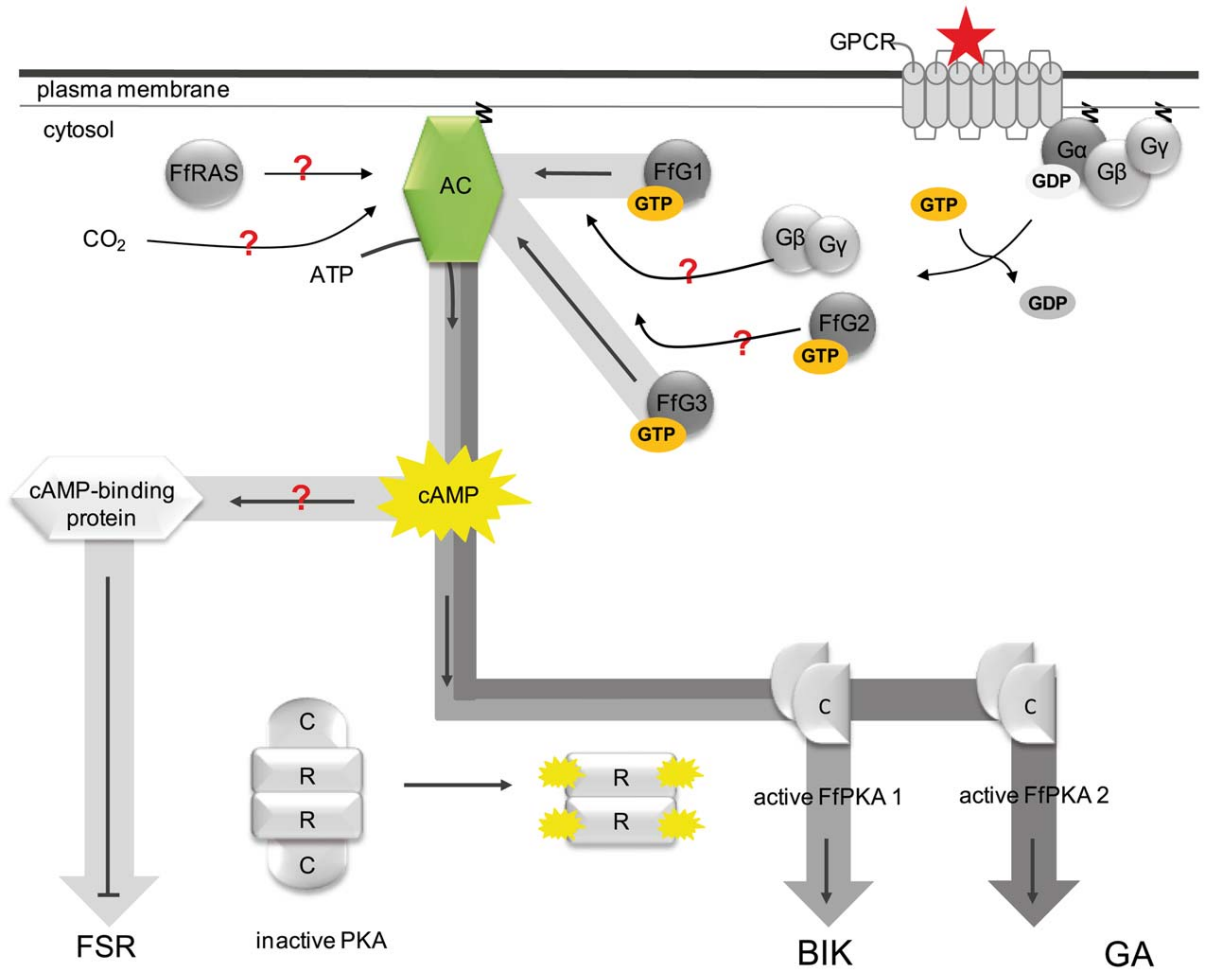
Involvement of G protein-mediated/cAMP signaling in the regulation of secondary metabolism in *F. fujikuroi*. Involvement of G protein-mediated/cAMP signaling in the regulation of secondary metabolism in *F. fujikuroi*. In the inactive state one GDP-bound Gα subunit (either one of FfG1, FfG2 and FfG3) is tightly associated with the Gβγ subunit forming a heterotrimeric G protein that is associated with a seven transmembrane helices-containing GPCR. In addition the Gα and Gγ subunits further anchor the heterotrimeric G protein to the plasma membrane through a N-terminal and a C-terminal acyl group, respectively. Ligand-binding to the GPCR leads to the exchange of GDP for GTP, resulting in a conformational change of the Gβγ-binding site and the dissociation of the Gα subunit from the Gβγ dimer. Each component (FfG1, FfG2, FfG3 or Gβγ) may now stimulate the downstream target, FfAC. FfAC in turn produces cAMP from ATP and activates the PKA. FfPKA1 then activates bikaverin biosynthesis, while FfPKA2 activates GA biosynthesis. Neither FfG1 nor FfG3 are involved in the regulation of GA biosynthesis. Whether FfG2, the monomeric G protein FfRAS or the CO_2_ level regulates GA biosynthesis via stimulation of cAMP accumulation awaits proof. Besides the PKA another cAMP-binding protein is most likely involved in the repression of fusarubin biosynthesis.

In conclusion, our studies describe the complex and diverse involvement of G protein/cAMP-mediated signaling in the regulation of fungal growth, secondary metabolism and sexual development in *F. fujikuroi*. We demonstrate that FfG1 (class I), FfG3 (class III) and FfAC negatively regulate the biosynthesis of fusarubin, probably by additive stimulating effects of FfAC by both Gα subunits. Surprisingly, neither FfPKA1 nor FfPKA2 have any impact on fusarubin production, indicating that another yet unidentified cAMP-binding protein exists that is involved in the regulation of the perithecial pigment. On the other hand, bikaverin production is regulated in an opposite way: its production is inhibited in the *ffg1*, *ffg3* and *ffac* deletion mutants. The opposite regulation of the two red pigments is also underlined by the contrasting effect of the global regulator Velvet: deletion of *ffvel1* resulted not only in increased bikaverin biosynthesis under both inducing and repressing conditions, but also in complete inhibition of fusarubin formation (fig. 8).

Although all three secondary metabolites, GA, fusarubin and bikaverin, depend on the activity of FfAC, only bikaverin and GA formation were shown to be regulated by the FfAC in a PKA-dependent manner. Furthermore, our results indicate that bikaverin biosynthesis is regulated by FfPKA1, while GA biosynthesis is regulated by FfPKA2. Previously we have demonstrated that GA and bikaverin biosynthesis, though both repressed by nitrogen, are regulated by contrasting nitrogen regulation pathways in an AreA-dependent and -independent manner, respectively, and that FfVel1 strongly represses bikaverin, but positively controls GAs formation [51]. All these data show the complexity of these regulatory networks, and that each secondary metabolite might be controlled by individual signaling pathways.

## Supporting Information

Figure S1 **Diagnostic PCR for Δ** ***ffg1*** **, Δ** ***ffg3,*** **Δ** ***ffac,*** **Δ** ***ffpka1,*** **Δ** ***ffpka2 and*** **MRC-1995/Δ** ***ffg1.*** Diagnostic PCR is shown for 5′, 3′ and for the detection of the wild-type gene (WT). A) Diagnostic PCR of the WT and two independent *ffg1* deletion mutants, Δ*ffg1_T9* and Δ*ffg1_T13.* B) Diagnostic PCR of the WT and four independent *ffpka2* deletion mutants: Δ*ffg3_T6*, Δ*ffg3_T7,* Δ*ffg3_T11* and Δ*ffg3_T12*. C) Diagnostic PCR of the WT and three independent deletion mutants for *ffac,* Δ*ffac_T7*, Δ*ffac_T12* and Δ*ffac_T14.* D) Diagnostic PCR of the WT and three independent *ffpka1* deletion mutants: Δ*ffpka1_T4*, Δ*ffpka1_T10* and Δ*ffpka1_T15.* E) Diagnostic PCR of the WT and four independent *ffpka2* deletion mutants: Δ*ffpka2_T10*, Δ*ffpka2_T12,* Δ*ffpka2_T15* and Δ*ffpka2_T16.* F). Diagnostic PCR of the WT and two independent *ffg1* deletion mutants in the *F. fujikuroi* wild type-strain MRC-1995: MRC-1995/Δ*ffg1_T44* and MRC-1995/Δ*ffg1_T49.*
(TIF)

Figure S2 **Strategy for constitutive expression of** ***ffg1*** **^G42R^ in** ***F. fujikuroi*** **.** Two fragments (I and II) were amplified from genomic DNA of IMI58289. Primers used for introduction of the point mutations are highlighted by orange bars. Plasmid containing the *ffg1* gene with the nucleotide substitution was transformed into the *F. fujikuroi* wild-type strain IMI58289. Homologous integration of the plasmid was verified by PCR and subsequent sequencing of the amplified fragment.
(TIF)

Figure S3 **Complementation of Δ** ***ffg1*** **, Δ** ***ffg3*** **and Δ** ***ffac.*** A) Complementation strategy. The gene of interest including ∼2-kb promoter sequence and ∼200-bp terminator sequence was amplified using proof-reading polymerase in two to five fragments. The fragments were transformed together with the SacII/SpeI-restriced plasmid NDN-OGG using yeast-recombinational cloning (for details see material and methods). Obtained clones were verified by Sequencing. Complementation was analyzed by plate assays. Therefore, the indicated strains were grown for five days on solidified ICI medium. B) plasmid for complementation and plate assay for Δ*ffg1/ffg1* ^C^. C) Plasmid for complementation and plate assay for Δ*ffac/ffac* ^C^. D) Plasmid for complementation and plate assay for Δ*ffg3/ffg3* ^C^.
(TIF)

Figure S4 **Complementation of Δ** ***ffg1*** **, Δ** ***ffg3*** **and Δ** ***ffac*** **restores the wild-type phenotype.** The indicated strains were grown for 4 days under bikaverin-favorable conditions (6 mM glutamine (Gln)). Culture filtrates were used for analysis of bikaverin and fusarubin accumulation by HPLC-DAD. The desired compounds were detected at 450 nm. Accumulation of bikaverin A) in the wild type (WT), B) in the deletion of *ffg1* and complementation thereof, C) in the deletion of *ffg3* and complementation thereof and D) in the deletion of *ffac* and complementation thereof.
(TIF)

Table S1 **Primers used during this study.**
(DOCX)
