# Supplementary figures and images for: Signaling Governed by G Proteins and cAMP Is Crucial for Growth, Secondary Metabolism and Sexual Development in Fusarium fujikuroi
Source: PLoS One. 2013 Feb 28;8(2):e58185. doi: 10.1371/journal.pone.0058185 (PMC3585259; doi:10.1371/journal.pone.0058185)

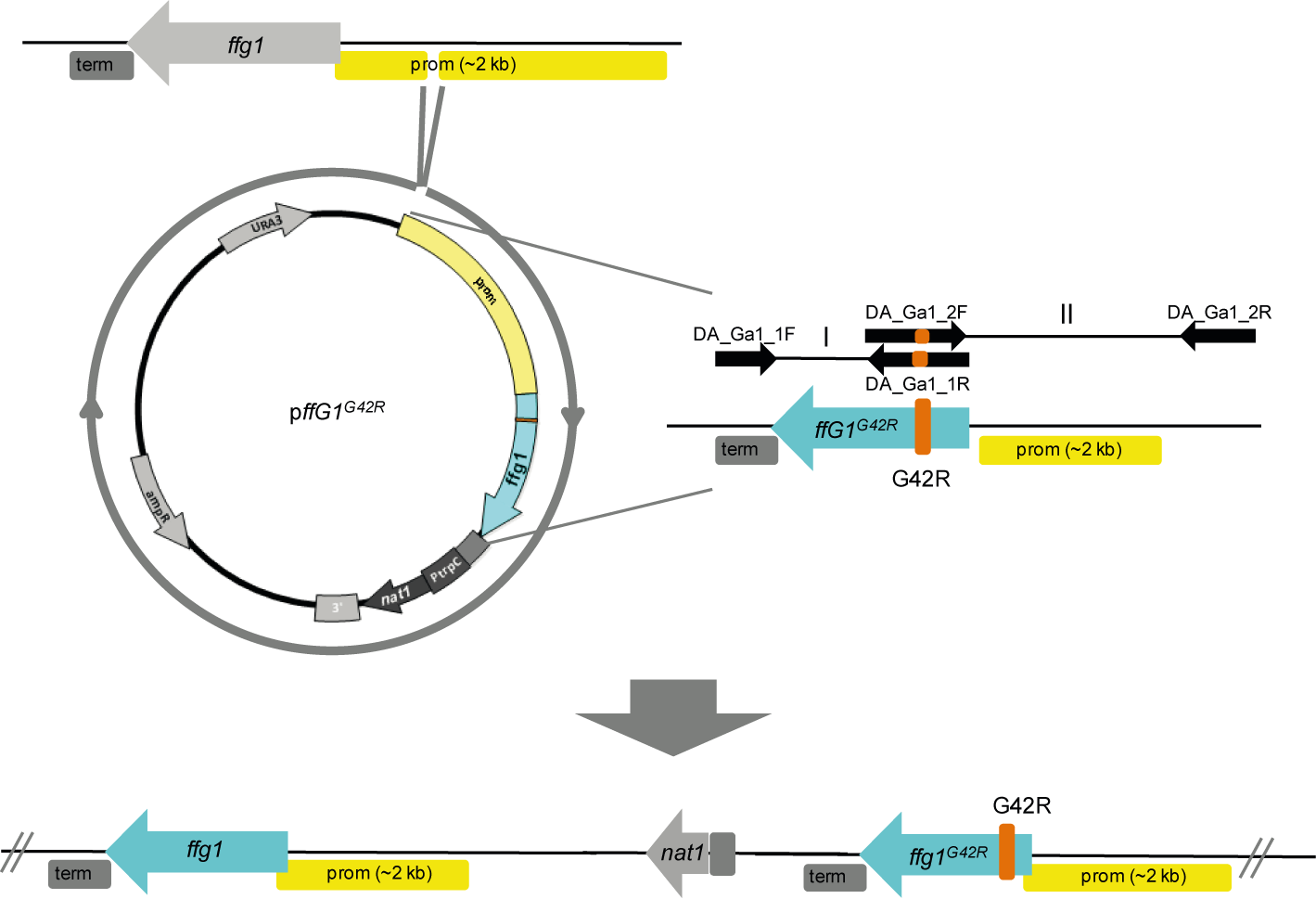

Supplement: Figure S2 — Strategy for constitutive expression of ffg1 G42R in F. fujikuroi . Two fragments (I and II) were amplified from genomic DNA of IMI58289. Primers used for introduction of the point mutations are highlighted by orange bars. Plasmid containing the ffg1 gene with the nucleotide substitution was transformed into the F. fujikuroi wild-type strain IMI58289. Homologous integration of the plasmid was verified by PCR and subsequent sequencing of the amplified fragment. (TIF) [file pone.0058185.s002.tif]

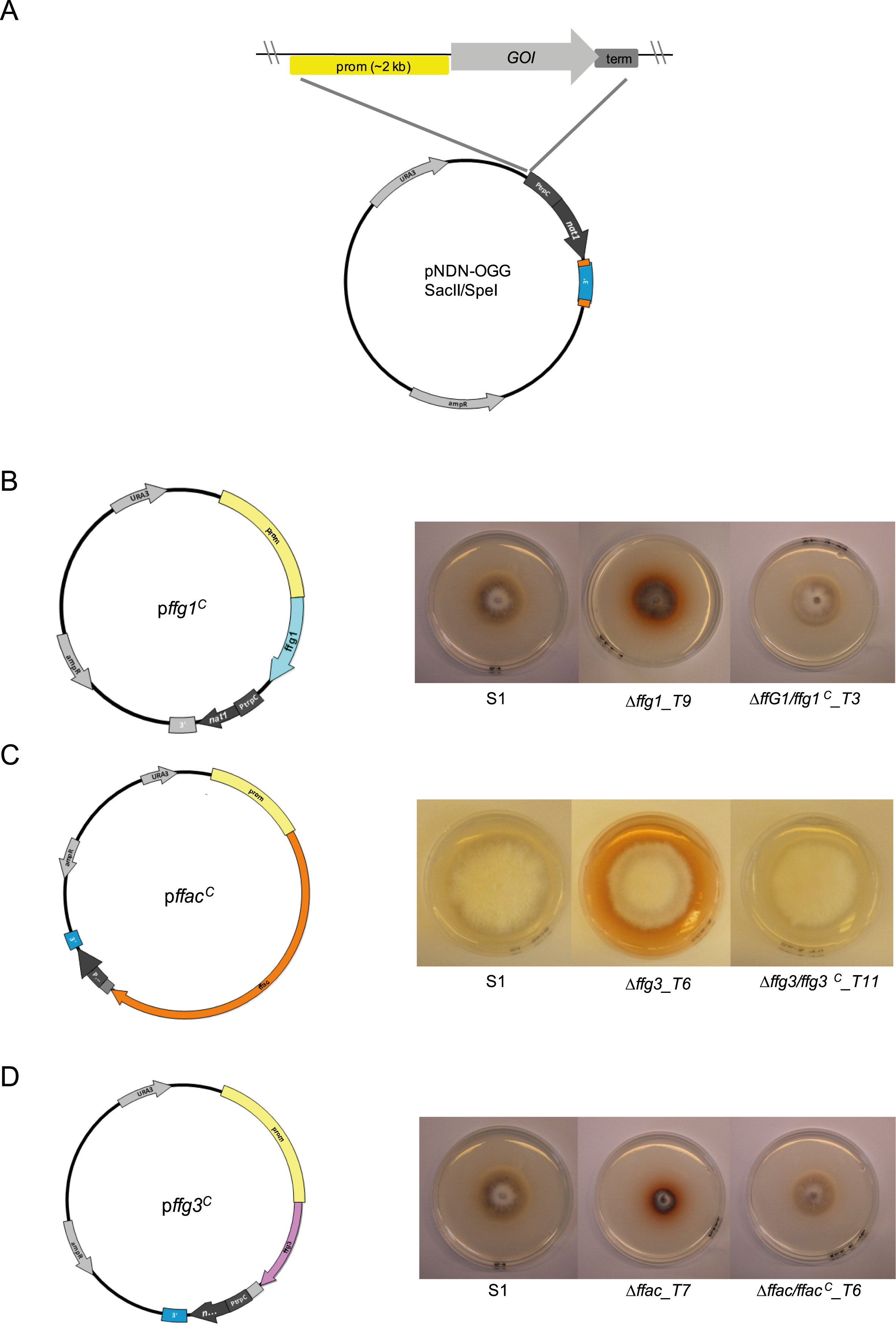

Supplement: Figure S3 — Complementation of Δ ffg1 , Δ ffg3 and Δ ffac. A) Complementation strategy. The gene of interest including ∼2-kb promoter sequence and ∼200-bp terminator sequence was amplified using proof-reading polymerase in two to five fragments. The fragments were transformed together with the SacII/SpeI-restriced plasmid NDN-OGG using yeast-recombinational cloning (for details see material and methods). Obtained clones were verified by Sequencing. Complementation was analyzed by plate assays. Therefore, the indicated strains were grown for five days on solidified ICI medium. B) plasmid for complementation and plate assay for Δffg1/ffg1 C. C) Plasmid for complementation and plate assay for Δffac/ffac C. D) Plasmid for complementation and plate assay for Δffg3/ffg3 C. (TIF) [file pone.0058185.s003.tif]

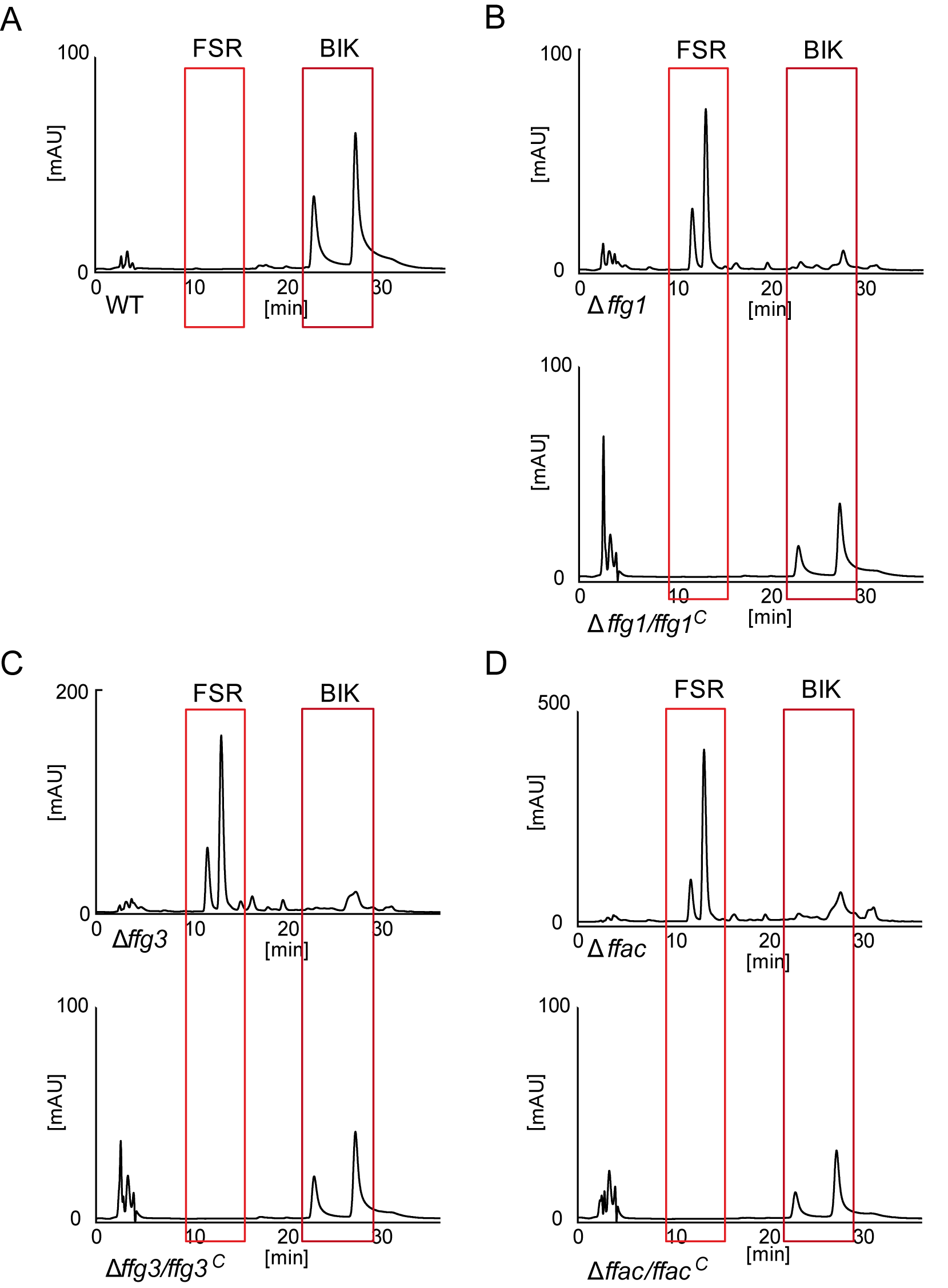

Supplement: Figure S4 — Complementation of Δ ffg1 , Δ ffg3 and Δ ffac restores the wild-type phenotype. The indicated strains were grown for 4 days under bikaverin-favorable conditions (6 mM glutamine (Gln)). Culture filtrates were used for analysis of bikaverin and fusarubin accumulation by HPLC-DAD. The desired compounds were detected at 450 nm. Accumulation of bikaverin A) in the wild type (WT), B) in the deletion of ffg1 and complementation thereof, C) in the deletion of ffg3 and complementation thereof and D) in the deletion of ffac and complementation thereof. (TIF) [file pone.0058185.s004.tif]
