# Supplementary material for: Signaling Governed by G Proteins and cAMP Is Crucial for Growth, Secondary Metabolism and Sexual Development in Fusarium fujikuroi
Source: PLoS One. 2013 Feb 28;8(2):e58185. doi: 10.1371/journal.pone.0058185 (PMC3585259; doi:10.1371/journal.pone.0058185)
Supplement: Table S1 — Primers used during this study. (DOCX) [file pone.0058185.s005.docx]

**Table S1** Primers used in this study

| gene | primer | primer sequence | |
| --- | --- | --- | --- |
| primers for knock out-design | | | |
| *ffg1* | Ffg1_5F | gtaacgccagggttttcccagtcacgacgTCTTGTATCCAATCAGCAGC | |
|  | Ffg1_5R | atccacttaacgttactgaaatctccaacGCTCCAACCTCTAGGTCTAGGG | |
|  | Ffg1_3F | ctccttcaatatcatcttctgtctccgacTTCGCTCCTCCGGTGTGACGAGTAC | |
|  | Ffg1_3R | gcggataacaatttcacacaggaaacagcACCTTCATAGTTCTGTAGGG | |
| *ffg3* | Ffg3_5F | gtaacgccagggttttcccagtcacgacgGAGTCAAACACGACAAGGTTTCG | |
|  | Ffg3_5R | atccacttaacgttactgaaatctccaacGAGCTGAGCACAAGTTCACTTC | |
|  | Ffg3_3F | ctccttcaatatcatcttctgtctccgacCACGAGACACCTGTACTTGGAGC | |
|  | Ffg3_3R | gcggataacaatttcacacaggaaacagcTACGTAGTGGAGGTAACACAGCG | |
| *ffac* | Ffac_5F | gtaacgccagggttttcccagtcacgacgTTTAGTACCTACGTAGCCTGCC | |
|  | Ffac_5R | atccacttaacgttactgaaatctccaacACTCATTCCTGGTCATTGTCGC | |
|  | Ffac_3F | ctccttcaatatcatcttctgtctccgacGAGTTGTGATTGGGTTCAGACG | |
|  | Ffac_3R | gcggataacaatttcacacaggaaacagcGACGTCTTGAGTTGTATGACGG | |
| *ffpka1* | Ffpka1_5_SacII | ATCCGCGGCAGTATCAGTATCAGTATCAGTCCC | |
|  | Ffpka1_5_XbaI | AATAGTGTGACGACAGATAGAGAGGG | |
|  | Ffpka1_3_SalI | GTCGACGAAAGTGCTTGAGAGCGAGTAATGG | |
|  | Ffpka1_3_XhoI | ATCTCGAGAGTGTGACAAGAAATGGACATCGGC | |
| *ffpka2* | Ffpka2_5F | gtaacgccagggttttcccagtcacgacgGAGTCCACTTTAAGTGCTCCG | |
|  | Ffpka2_5R | atccacttaacgttactgaaatctccaacGTCCTGAAGTCTTGTGATAGG | |
|  | Ffpka2_3F | ctccttcaatatcatcttctgtctccgacACACGGCAGTCAGACTCTACC | |
|  | Ffpka2_3R | gcggataacaatttcacacaggaaacagcTTCCTACGTATGGACGTGCC | |
| *hph* | hph_F | GTCGGAGACAGAAGATGATATTGAAGGAGC | |
|  | hph_R | GTTGGAGATTTCAGTAACGTTAAGTGGAT | |
| Diagnostische PCR_KO | | | |
| *ffg1* | Dia_Ga1_5‘ | TGAAACCATCTTCCACTCACC | |
|  | Dia-Ga1_3‘ | TCGAGTCAAGTCACATGACC | |
|  | Dia_Ga1_WT_F | CGAGATCAAGATGCTCCTGC | |
|  | Dia_Ga1_WT_R | AATCGAACAGAGTGAGAGCC | |
| *ffg3* | Dia_Ga3_5‘ | ATTCCGTAGGTGCTCAGTGC | |
|  | Dia-Ga3_3‘ | GACAGCTGATGCTTGCATCG | |
|  | Dia_Ga3_WT_F | CAACAATTCGACATCGAGCC | |
|  | Dia_Ga3_WT_R | TGACGTATCTGTTGCTTGCG | |
| *ffac* | Dia_ffac_5‘ | GGCGTAACAGTGAACCTACAGTACC | |
|  | Dia_ffac_3‘ | GATGACCCTACTCCTGTCATCGACG | |
|  | Dia_ffac_WT_F | CCACCTCTAATACCTCGAC | |
|  | Dia_ffac_WT_R | CGGAGAGGAATGTGTCGATC | |
| *ffpka1* | Dia_ffpka1_5‘ | TTGGACACGAACCGACACAAGGC | |
|  | Dia_ffpka1_3‘ | GGTCCCTACCTACGGATACTCAGG | |
|  | Dia_ffpka1_WT_F | ACACGGCAGTCAGACTCTACC | |
|  | Dia_ffpka1_WT_R | TTCCTACGTATGGACGTGCC | |
| *ffpka2* | Dia_ffpka2_5‘ | CTGACAACTTGTCGATCGC | |
|  | Dia_ffpka2_3‘ | CGATGTGCTACCACCACGACC | |
|  | Dia_ffpka2_WT_F | ATTCTCGCTGATGTCGCAGG | |
|  | Dia_ffpka2_WT_R | GTCGAAGCATTGTGCATCG | |
| trpC-T | pCSN44-hph-trpC-T | GGAATAGAGTAGATGCCGACCGG | |
| trpC-P | pCSN44-trpC-P | CCTCCACTAGCTCCAGCCAAGCCC | |
| Primers for creation of complementation vectors and constitutive FfG1 | | | |
| *ffg1^C^* | Com_Ga1_1F | gtaacgccagggttttcccagtcacgacgccgcCTTTCACTCTGGTGAGTAGCCAGCCAG | |
|  | Com_Ga1_1R | TCCAGATTCACCAGCACCTGTGAGC | |
|  | Com_Ga1_2F | GCTCACAGGTGCTGGTGAATCTGGA | |
|  | Com_Ga1_2R | gcccaaaaaatgctccttcaatgtcactagCAGCCAAAGCTAGTGGTAAATCTGGCG | |
| *ffg1*^G42R^ | DA_Ga1_1F | gtaacgccagggttttcccagtcacgacgccgcCTTTCACTCTGGTGAGTAGCCAGCCAG | |
|  | DA_Ga1_1R | TCCAGATTCACgAGCACCTGTGAGC | |
|  | DA_Ga1_2F | GCTCACAGGTGCTcGTGAATCTGGA | |
|  | DA_Ga1_2R | gcccaaaaaatgctccttcaatgtcactagCAGCCAAAGCTAGTGGTAAATCTGGCG | |
|  |  |  | |
| **Table S1** (continued) Primers used in this study | | | |
| Primers for creation of complementation vectors and constitutive FfG1 | | |  |
| *ffg3^C^* | Com_Ga-3_1F | ggttttcccagtcacgacgccgcGATGGCAGCAAGTCTTGGTCTACTAGG |  |
|  | Com-Ga-3_1R | CTCATTGCTCGAGCTCATGCATGC |  |
|  | Com-Ga3_2F | GTGAACTTGTGCTCAGCTCACCG |  |
|  | Com-Ga3_2R | ccaaaaaatgctccttcaatgtcactagGAGACTGGCACAAGAGAAATGC |  |
| *ffac^C^* | Com_ffac_1F | agggttttcccagtcacgacgccgcGGACCGACCATAGGATTGTGGCACG |  |
|  | Com_ffac_1R | CAGTTTGGTTCACAGGTGCC |  |
|  | Com_ffac_2F | AACGACATACCTCACCTTTCCG |  |
|  | Com_ffac_2R | CCAGCACGCTAGTGAGAATGC |  |
|  | Com_ffac_3F | GATCGACACATTCCTCTCCG |  |
|  | Com_ffac_3R | TGATGTCGATGCTCGAGATACC |  |
|  | Com_ffac_4F | CAACGATTCCTCACGAGATTGG |  |
|  | Com_ffac_4R | ACCATCTGTCTGGATCACCATCG |  |
|  | Com_ffac_5F | ACCAAGGAAGACCTCAACTCAGG |  |
|  | Com_ffac_5R | caaaaaatgctccttcaatgtcactagCAAACACATGAGCACATCGCACC |  |
| Primers for plasmid sequencing | | |  |
| *ffg1^C^/*  *ffg1^G42R^* | Seq_Ga1_1 | CTCTTCGCTATTACGCCAGC |  |
|  | Seq_Ga1_2 | TGAAACCATCTTCCACTCACC |  |
|  | Seq_Ga1_3 | CTGTGATTACTGCTTTGTGCC |  |
|  | Seq_Ga1_4 | GCTCCAACCTCTAGGTCTAGG |  |
|  | Seq_Ga1_5 | GTTAATCACTAACGTCGACGG |  |
|  | Seq_Ga1_6 | ACTTCGACAACATTGCTCGC |  |
|  | Seq_Ga1_7 | GGCTTGCGATTACATCCTCA |  |
|  | Seq_Ga1_8 | TTGACCTCCACTAGCTCCAGC |  |
| *ffg3^C^* | Seq_Ga3_Com_1 | AGCCAAACAAATCAGAAGGG |  |
|  | Seq_Ga3_Com_2 | ATTCCGTAGGTGCTCAGTGC |  |
|  | Seq_Ga3_Com_3 | CTCGTCATCGTCGAGGTTTCC |  |
|  | Seq_Ga3_Com_4 | GCAAATCGATTCGAAAGTGG |  |
|  | Seq_Ga3_Com_5 | GAGCAGTCAGGTACGCTTGG |  |
| *ffac^C^* | Seq_ffac_Com_1 | CATTCGCCATTCAGGCTGC |  |
|  | Seq_ffac_Com_2 | GCGAGATGAGAAGATCCACG |  |
|  | Seq_ffac_Com_3 | TTACCTACACTGTACTGCC |  |
|  | Seq_ffac_Com_4 | ACAGCAATGATGACAGTCCG |  |
|  | Seq_ffac_Com_5 | AGGAATGACTTGGAGCACG |  |
|  | Seq_ffac_Com_6 | TCTCGATACCAACCTGAACG |  |
|  | Seq_ffac_Com_7 | GACTCCTGCAGCAAGTCGG |  |
|  | Seq_ffac_Com_8 | AATTCCGTTGGAGAGCTACC |  |
|  | Seq_ffac_Com_9 | CAACGCAAACTGTCAACACC |  |
|  | Seq_ffac_Com_10 | GATACCTGAATCTGTCTGG |  |
|  | Seq_ffac_Com_11 | ATGATTAGTGTTGCTGACC |  |
|  | Seq_ffac_Com_12 | GCTCCTCGATGTGAACTGG |  |
|  | Seq_ffac_Com_13 | TATCGAATTCCACCTGCAGC |  |
|  | Seq_ffac_Com_14 | ACACGAGGAACACGACAAGG |  |

*Note: Sequences complementary to pRS426, pNDN and resistance cassettes are written in small letters.*
